# The TRAX, DISC1, and GSK3 complex in mental disorders and therapeutic interventions

**DOI:** 10.1186/s12929-018-0473-x

**Published:** 2018-10-04

**Authors:** Yu-Ting Weng, Ting Chien, I-I Kuan, Yijuang Chern

**Affiliations:** 1 Institute of Biomedical Sciences, Academia Sinica, 128 Sec. 2, Academia Rd. Nankang, Taipei, 115 Taiwan, Republic of China; 2 Program in Molecular Medicine, National Yang-Ming University and Academia Sinica, No.155, Sec.2, Linong Street, Taipei, 112 Taiwan, Republic of China

**Keywords:** TRAX, DISC1, GSK3β, Mental disorders, DNA damage, DNA repair, Oxidative stress, A_2A_R, PKA

## Abstract

Psychiatric disorders (such as bipolar disorder, depression, and schizophrenia) affect the lives of millions of individuals worldwide. Despite the tremendous efforts devoted to various types of psychiatric studies and rapidly accumulating genetic information, the molecular mechanisms underlying psychiatric disorder development remain elusive. Among the genes that have been implicated in schizophrenia and other mental disorders, disrupted in schizophrenia 1 (DISC1) and glycogen synthase kinase 3 (GSK3) have been intensively investigated. DISC1 binds directly to GSK3 and modulates many cellular functions by negatively inhibiting GSK3 activity. The human DISC1 gene is located on chromosome 1 and is highly associated with schizophrenia and other mental disorders. A recent study demonstrated that a neighboring gene of DISC1, translin-associated factor X (TRAX), binds to the DISC1/GSK3β complex and at least partly mediates the actions of the DISC1/GSK3β complex. Previous studies also demonstrate that TRAX and most of its interacting proteins that have been identified so far are risk genes and/or markers of mental disorders. In the present review, we will focus on the emerging roles of TRAX and its interacting proteins (including DISC1 and GSK3β) in psychiatric disorders and the potential implications for developing therapeutic interventions.

## Background

Mental disorders (such as bipolar disorder, depression, and schizophrenia) have recently become great concerns because of the resultant heavy social and economic burdens on societies [1–3]. Rapidly progressing genetic technologies have provided many details regarding the genetic nature of mental disorders. Among the genes that have been revealed by genetic analyses of schizophrenia and other mental disorders, the function of disrupted in schizophrenia 1 (DISC1) has been intensively investigated. Biochemical investigations suggest that DISC1 is a scaffold protein that regulates various cellular functions (including cytoskeletal processes, intracellular transport, dendritic spine development activities, neuronal development, the cAMP-signaling pathway, and DNA repair) by interacting with various proteins [4–12]. Thus, DISC1 has been considered as a hub protein for schizophrenia and possibly other mental diseases (Table 1).

**Table 1:** Potential involvement of TRAX-interacting proteins in three psychiatric disorders

Binding partner	Full name	Gene name	Schizophrenia	Autism	Panic attack
A_2A_R [5, 132, 134]	A_2A_ adenosine receptor	ADORA2A	Drug target [164–166]	Risk gene (#)	Risk gene [167]
Akap9 [168]	A-kinase anchoring protein 9	AKAP9	Risk gene [169, 170]	Risk gene (#, [171–173])	Risk gene [167]
ATM [137]	Ataxia telangiectasia mutated	ATM	Risk gene [139, 174]	–	–
C1D [136]	nuclear matrix protein C1D	C1D	(1) Risk gene (*)	–	–
			(2) Drug target [175]		
DISC1 [5]	Disrupted in schizophrenia 1	DISC1	(1) Risk gene (*)	Risk gene [176, 177]	Risk gene [167]
			(2) Drug target [116, 178]		
GSK3β [5]	Glycogen Synthase Kinase 3 Beta	GSK3B	(1) Risk gene (*)	Risk gene [179, 180]	Risk gene [181]
			(2) Drug target [182]		
KIF2A [134, 183]	Kinesin Family Member 2A	KIF2A	Risk gene (*)	–	–
MEA2 [168]	Male-enhanced antigen 2	MEA2	–	–	–
PLCβ1 [184, 185]	Phospholipase C Beta 1	PLCB1	Risk gene (*, [185–187])	Risk gene (#, [188])	–
SUN1 [168]	SUN domain-containing protein 1	SUN1	–	–	–
Translin [126–128, 130, 189, 190]	Translin	TSN	Risk gene [191, 192]	Risk gene (#, [193])	–
TRAX-interacting protein-1 [22]	Translin Associated Factor X Interacting Protein 1	TSNAXIP1	Risk gene [194]	–	–

The corresponding references are listed in parentheses. “-”, no information. *, http://www.szdb.org/score.php. #, https://gene.sfari.org/database/human-gene/

Previous genetic studies have associated *DISC1* and a neighboring gene (*translin-associated factor X*, *TSNAX*) with multiple mental disorders (e.g., schizophrenia, bipolar spectrum disorder, and major depressive disorder) [13–15] (Table 1). TRAX was initially identified as a binding partner of an RNA/DNA-binding protein (translin [16]). Further investigations revealed that similar to DISC1, TRAX regulates distinct cellular functions by selectively binding to designated partner(s). Moreover, the list of TRAX-interacting proteins overlaps with that of DISC1 (Table 2). Both TRAX and DISC1 are involved in facilitating DNA repair [5]. Chien et al. demonstrated that TRAX forms a complex with DISC1 and GSK3β in the cytoplasmic region of resting neurons. Upon stresses that cause oxidative DNA damage, inhibiting GSK3β causes the TRAX/DISC1/GSK3β complex to dissociate and release TRAX to facilitate ATM-mediated DNA repair [5]. Because the incomplete repair of oxidative DNA damage may contribute to the development of psychotic disorders [1, 17, 18] and because TRAX and many of its interacting proteins (Table 1) are risk genes and/or markers of mental disorders, the present review focuses on the emerging role of TRAX/DISC1 interactome(s) in DNA repair as well as their potential implications in psychiatric disorders.

**Table 2:** Pathways interacting with DISC1 and/or TRAX

Pathway	Binding partner	Full name	Interaction with TRAX	Interaction with DISC1
cAMP/PKA	A_2A_R [5, 132, 134]	A_2A_ adenosine receptor	+ [132]	Nd
	Akap9 [168]	A-Kinase Anchoring Protein 9	+ [168]	+ [194, 195]
	ATF4	Activating Transcription Factor 4	nd	+ [194, 196, 197]
	ATF5	Activating Transcription Factor 5	nd	+ [194, 198–200]
	ATF7IP	Activating Transcription Factor 7	nd	+ [194]
	D2R	Dopamine D2 receptor	nd	+ [146]
	PDE4B	Phosphodiesterase 4B	nd	+ [8, 194, 201]
	PDE4D	Phosphodiesterase 4D	nd	+ [194, 202]
Wnt signaling	GSK3β [5]	Glycogen Synthase Kinase 3 β	+ [5]	+ [5, 7, 194]
	β-catenin	Catenin β-1	nd	+ [7, 194, 203]
	DIXDC1	DIX Domain Containing 1	nd	+ [194, 204]
	TNIK	TRAF2 And NCK Interacting Kinase	nd	+ [194, 205, 206]
	WNT3A	Wnt Family Member 3A	nd	+ [194]
Intracellular Transport	Dynactin	Dynactin	nd	+ [207]
	FEZ1	Fasciculation And Elongation Protein Zeta 1	nd	+ [208, 209]
	HZF	Haematopoetic zinc finger	nd	+ [12]
	KIF1B	Kinesin Family Member 1B	nd	+ [12]
	KIF2A	Kinesin Family Member 2A	+ [134, 183]	nd
	KIF5A	Kinesin Family Member 5A	nd	+ [11, 12]
	Miro1/2	Mitochondrial Rho GTPase 1/2	nd	+ [9, 210]
	SNPH	Syntaphilin	nd	+ [10]
	TRAK1/2	Trafficking kinesin protein-1/2	nd	+ [9]
	Translin	Tanslin	+ [211]	nd
DNA repair	ATM	ataxia-telangiectasia mutated	+ [137]	nd
	C1D	nuclear matrix protein C1D	+ [136]	nd
	Rad21	Double-strand-break repair protein rad21 homolog	Nd	+ [212, 213]

Accumulating evidence suggests the involvement of DISC1/TRAX in several signaling pathways and machineries that mediate a wide variety of cellular functions. +, direct interaction. nd, not determined. The corresponding references are listed in parentheses

### DNA damage, oxidative stress, and mental health

Reactive oxygen species (ROS) are usually generated through mitochondrial oxidative reactions [19]. Excessive ROS levels are a source of oxidative stress, which causes oxidative damage to DNA, proteins and lipids. ROS can attack the nitrogenous bases and sugar-phosphate backbone of DNA to cause single- and double-stranded DNA breaks that ultimately lead to genetic mutations and toxicity [20]. When cells are subjected to increased levels of ROS and reactive nitrogen species, multiple cellular impairments (e.g., oxidative DNA damage) occur [21]. Accumulating evidence suggests that elevated ROS levels and the resultant oxidative damage are major factors in human health and diseases [21–24]. Because the brain uses approximately 20% of the total oxygen in the body and generates significant amounts of free radicals, the brain is more susceptible to oxidative stress than other organs. Moreover, elevated ROS levels have been implicated in most neurological diseases (such as mental disorder and neurodegenerative diseases) [25–27]. Elevated levels of serum oxidative markers (such as 8-hydroxy-2′-deoxyguanosine, 8-OHdG) have also been reported in patients with trauma or diseases of the brain [28–31].

Ample evidence suggests that increased oxidative stress, which may cause oxidative DNA damage and mitochondrial dysfunction, is a common feature of mental disorders in the brain. Mitochondrial dysfunction is associated directly with elevated levels of oxidative stress and the progression of mental disorders [18, 19, 32]. For example, the nuclear gene expression levels of mitochondrial proteins, including electron transport chain (ETC) complexes I–V, are significantly decreased in the hippocampus and postmortem frontal cortex of patients with bipolar disorder and schizophrenia [33–35]. It is important to note that ETC complex I is one of the major sources of ROS in mitochondria. Moreover, the expression levels of NADH:Ubiquinone oxidoreductase core subunit v2 (NDUFV2), a mitochondrial complex I subunit gene, were decreased in lymphoblastoid cell lines derived from patients with bipolar disorder [36]. These findings indicate that mitochondria dysfunction is a major factor that contributes to the development of mental disorders, including bipolar disorder and schizophrenia [19]. Another important feature of the brains of patients with mental disorders is an imbalance in the levels of dopamine and glutamate (for a review, see [37]). Accumulating evidence suggests that hypofunction of NMDA receptors was observed in schizophrenia [38, 39]. Several NMDA receptor antagonists (e.g., phencyclidine and ketamine) therefore have been shown to induce schizophrenia-like symptoms [40, 41]. Other studies reported that hypofunction of synaptic NMDA receptors are detrimental to neurons. Activation of synaptic NMDA receptors promotes signaling pathways that have been implicated in neuronal survival [42]. Thus, the enhancement of NMDA receptor function may serve as a potential therapeutic strategy for patient with schizophrenia. It should be noted that excess glutamate causes calcium influx and subsequently facilitates the generation of ROS [43, 44].

In addition to high oxidative stress levels, impaired DNA repair is also a pathogenic feature of mental disorders [1]. Many genes involved in DNA repair or DNA damage detection have also been implicated in mental disorders. For example, variants of genes involved in DNA repair, such as x-ray repair cross complementing 1 (XRCC1), XRCC3, human 8-oxoguanine DNA N-glycosylase 1 (hOGG1), and xeroderma pigmentosum group D (XPD), have been documented in schizophrenia pathophysiology [2]. Improving DNA repair is thus a possible strategy for developing therapeutic interventions for mental disorders. In the present review, the emerging role of a new set of risk genes (DISC1, GSK3β, and TRAX) for mental disorders in the repair of oxidative DNA damage will be discussed.

### GSK3

GSK3 was originally identified as a highly specific serine/threonine kinase for glycogen synthase in rabbit skeletal muscle [45]. There are two types of GSK3, GSK3α and GSK3β, and these are encoded by two different genes that share 83% identity in humans [46]. GSK3 activity can be regulated positively by the phosphorylation of GSK3α and GSK3β at Tyr^279^ and Tyr^216^, respectively [47], and negatively by the phosphorylation of GSK3α and GSK3β at Ser^21^ and Ser^9^, respectively [48, 49]. The phosphorylation of Tyr^279^-GSK3α and Tyr^216^-GSK3β are intramolecular autophosphorylation events [50], whereas the phosphorylation of Ser^21^-GSK3α and Ser^9^-GSK3β can be mediated by several kinases, including AKT [51] and protein kinase A (PKA) [52]. Both GSK3α and GSK3β are expressed highly in the mouse brain [53], whereas GSK3β is mainly expressed in the human brain [54]. GSK3β is thus expected to play a critical role in the brain.

As a kinase, GSK3β is involved in diverse biological activities and pathways by phosphorylating its downstream substrates. Briefly, GSK3β regulates neurite outgrowth, neuronal polarization and microtubule dynamics by phosphorylating several microtubule-associated proteins (MAPs), such as tau [55], MAP1β [56] and collapsin response mediator protein-2 (CRMP-2) [57]. GSK3β also regulates structural synaptic plasticity. GSK3β phosphorylates β-catenin and promotes β-catenin degradation [58]. GSK3β deletion in a subset of cortical and hippocampal neurons results in constitutively active β-catenin, which reduces spine density and excitatory synaptic neurotransmission [59]. GSK3β deletion in dentate gyrus (DG) excitatory neurons also reduces the levels of several synaptic proteins and subunits of N-methyl-D-aspartate (NMDA) and α-amino-3-hydroxy-5-methyl-4- isoxazolepropionic acid (AMPA) receptors and inhibits calcium/calmodulin-dependent protein kinase II (CaMKII)/CaMKIV-cAMP response element binding protein (CREB) signaling [60]. Furthermore, GSK3β is involved in long-term potentiation (LTP) and long-term depression (LTD). During LTP induction in the DG and CA1 areas of the hippocampus, the phosphorylation level of GSK3β at Ser^9^ is increased, which subsequently inhibits the induction of LTD [61–63]. GSK3β overexpression in the hippocampus reduces neurotransmitter release and hyper-phosphorylates tau, which impairs the induction of LTP and learning [64, 65]. In addition, GSK3 inhibition rescues the number of abnormal dendritic spines and glutamatergic synapses in pyramidal neurons and may improve the psychiatric pathogenesis caused by DIXDC1/GSK3 axis impairment in mental disorders [66].

GSK3β is also involved in apoptotic regulation in response to several stresses, including DNA damage [67] and oxidative stress [68]. In response to DNA damage, the interaction between GSK3β and p53 enhances the activity of GSK3β and p53-mediated apoptosis via increasing p21 protein levels and caspase-3 activation [67]. GSK3β inactivation protects hippocampal [69] and cerebellar granule neurons [70] from irradiation-induced death through inhibiting p53 accumulation [71]. In neurons, oxidative stress exposure for a short period of time reduces the activity of GSK3β, while prolonged exposure to ROS increases GSK3β activity [72, 73]. Therefore, GSK3β is a redox-sensitive kinase. GSK3β activation in response to oxidative stress downregulates the nuclear-localized NF-E2-related factor 2 (NRF2), which inhibits the expression of antioxidant genes, such as heme oxygenase-1 (HO-1), and sensitizes neurons to oxidative stress-induced death [72]. Furthermore, GSK3β activation in response to oxidative stress phosphorylates and induces the degradation of CRMP-2, a cytoskeleton regulator involved in lithium response in bipolar disorder patients [74], and results in axonal degeneration and neuronal death [73, 75]. GSK3β inhibition is thus expected to protect neurons from oxidative stress-induced damage and death. Consistently, GSK3β inhibition through activating A_2A_ adenosine receptor (A_2a_R) has protective effects on oxidative stress-induced DNA damage because its binding partner (TRAX) is released to facilitate DNA repair and improve survival [5].

Accumulating evidence suggests that the dysregulation of GSK3β and/or its up/downstream molecules may contribute to bipolar disorder and schizophrenia. The inhibitory phosphorylation levels of GSK3 are lower in the peripheral blood mononuclear cells (PBMCs) from bipolar disorder patients than in those from healthy controls [76], but not in platelets [77]. Interestingly, although the protein levels of GSK3 are higher in PBMCs from type 1 bipolar disorder patients than in those from normal subjects, the amount of inhibitory GSK3 phosphorylation shows only a decreasing trend [78]. Conversely, the protein levels of GSK3β in the frontal cortex and cerebrospinal fluid are lower in schizophrenia patients than in normal subjects [79, 80]. However, other studies have failed to show changes in the protein levels or activity of GSK3β in patients with mental diseases compared to normal controls [81, 82].

One reason that GSK3β is linked to psychiatric diseases is that GSK3 is a target of lithium, a mood stabilizer used to treat mental diseases [83]. Lithium enhances the phosphorylation of GSK3β at Ser^9^ to inhibit GSK3β directly through competition with magnesium [84] and indirectly by activating AKT [85]. Significant efforts have thus been devoted to the design and development of new GSK3 inhibitors [86–89]. Several new GSK3 inhibitors have been assessed in mouse models of bipolar diseases. For example, the maleimide derivative, 3-(Benzofuran-3-yl)-4-(indol-3-yl)maleimide compound 2B, which mimics the structure of lithium, inhibits GSK3β activity and locomotor hyperactivity induced by the combination of amphetamine and chlordiazepoxide as a model for the manic phase of bipolar disease [90]. Additional GSK3 inhibitors (including indirubin, alsterpaullone, TDZD-8, AR-A014418, SB-216763, and SB-627772) were shown to inhibit rearing hyperactivity in the amphetamine-induced hyperactivity [91]. In the present review, we focus on a novel function of GSK3 that may provide new insights into the role of GSK3 in neuronal development and psychiatric pathogenesis.

### DISC1

DISC1 was initially identified in a large Scottish family with a spectrum of mental diseases (including schizophrenia, recurrent major depression and bipolar disorder) [92–95]. The N-terminal globular domain of DISC1 contains a conserved nuclear localization signal, and the C-terminal coiled-coil region is predicted to mediate its interactions with different proteins [96]. DISC1 is highly expressed in the heart, brain and placenta of humans [94] and in the heart, brain, kidney, and testis of mice [97]. DISC1 expression in the brain is regulated during development; its highest level occurs during the neonatal-infancy period and decreases gradually with age in human brains [98]. It is important to note that DISC1 expression may be regulated by environmental stimuli too. For example, the activation of Toll-like receptor 3 (TLR3) during viral infection leads to the downregulation of DISC1 through myeloid differentiation primary response gene 88 (MYD88) and subsequently impairs dendritic arborization and neuronal development [99]. Such cytoarchitectural defects (e.g., dendritic organization) have been found in schizophrenic subjects [100, 101], suggesting the importance of DISC1 in the regulation of neuronal development at prenatal and neonatal stages. Given the correlation between the deficits in neuronal development and the risk of developing schizophrenia, schizophrenia is also referred to as a neurodevelopmental disorder.

Previous studies suggest that DISC1 functions as a scaffold protein and mediates diverse neurodevelopmental processes by interacting with different proteins (Table 2). Specifically, DISC1 regulates cytoskeletal processes (e.g., neurite outgrowth and neuronal migration) by interacting with several proteins that are localized to the centrosome and axonal growth cones, including lissencephaly 1 (LIS1), nuclear distribution nudE-Like 1 (NDEL1) [11, 102], NDE1 [103], pericentriolar material 1 (PCM1), and Bardet-Biedl syndrome 4 (BBS4) [104].Given that DISC1 is located at the post-synaptic density (PSD) in the human neocortex [105], DISC1 is likely to play an important role in dendritic spine development and synaptic activities. DISC1 interacts with kalirin-7 (kal-7) at the glutamatergic PSD and mediates the interaction between kal-7 and PSD-95 or Rac family small GTPase 1 (Rac1) to regulate the size and number of spines [6].

Another important function of DISC1 is its regulation of the cyclic adenosine monophosphate (cAMP)-signaling pathway by binding and inhibiting phosphodiesterase 4B (PDE4B; Table 2). Increased cAMP levels cause DISC1 and PDE4B dissociation and enhance PDE4B activity [8]. The DISC1/PDE4 complex also regulates the PKA-mediated phosphorylation and association of a complex (NDE1/LIS1/NDEL1) important for neuronal development [106]. In addition, DISC1 interacts with several key molecules in the cAMP/PKA pathway, including an anchoring protein of PKA (A-kinase anchoring protein 9 (AKAP9); Table 2), several transcription factors (activating transcription factor 4 and 5 (ATF4 and ATF5); Table 2) that recognize the cAMP response element, and a Giα-coupled receptor that suppresses cAMP production upon activation (D2 dopamine receptor (D2R); Table 2).

DISC1 also plays an important role in intracellular transport (Table 2). By interacting with syntaphilin (SNPH), Mitochondrial Rho GTPase 1/2 (Miro1/2), and Trafficking kinesin protein-1/2 (TRAK1/2), DISC1 mediates the transport of mitochondria in the axons and dendrites [4, 9, 10]. DISC1 is involved in the transport of synaptic vesicles because it stabilizes the interaction between fasciculation and elongation protein zeta 1 (FEZ1) and synaptotagmin-1 (SYT-1) in the axons [107]. Moreover, DISC1 interacts with hematopoietic zinc finger (HZF) to mediate the dendritic transport of *inositol-1,4,5-trisphosphate receptor type 1(ITRP1)* mRNA [12].

Another important interacting protein of DISC1 is GSK3β, as well as several proteins involved in the Wnt pathway (Table 2). The direct binding of DISC1 inhibits GSK3β activity [7]. The interaction between DISC1 and GSK3β controls the fate of neural progenitors in the ventricular zone/subventricular zone [7] and subgranular zone of the dentate gyrus [97]. GSK3β inhibition prevents the phosphorylation and degradation of β-catenin, its downstream target [58], resulting in increased neural progenitor proliferation [108]. Intriguingly, the phosphorylation of DISC1 at Ser^710^ determines the affinity of DISC1 toward its binding partners. For example, non-phosphorylated DISC1 at Ser^710^ inhibits GSK3β and subsequently activates β-catenin signaling. Conversely, the phosphorylation of DISC1 at Ser^710^ increases the affinity of DISC1 for another binding partner, BBS protein, which facilitates the recruitment of BBS to the centrosome and subsequently causes the transition from progenitor proliferation to neuronal migration in the developing cortex [109]. It is interesting to note that the PKA-mediated inhibitory phosphorylation of GSK3β at Ser^9^ leads to the dissociation of the DISC1/GSK3β/TRAX complex and facilitates TRAX-mediated DNA repair in neurons [5]. These results collectively suggest that phosphorylation is a key modulatory mechanism that regulates the complex formation of DISC1 and other interaction proteins through which a wide variety of cellular functions are regulated.

Ample genetic evidence links DISC1 with major mental illnesses. The balanced (1;11) translocation of DISC1 within a Scottish family increased the incidence of major mental illnesses [95], probably due to the decrease in DISC1 protein levels [8] or the production of a dominant-negative C-terminal truncated DISC1 that loses its interaction with DISC1-interacting proteins [110, 111]. In addition, expression levels of the DISC1-interacting proteins LIS1 and NDEL1 are decreased in the brains of schizophrenia patients and are associated with high-risk DISC1 SNPs [98]. To date, the DISC1 gene has been identified as a risk factor for major mental illnesses [13, 112–120]. In contrast, some other reports have failed to show the association between DISC1 variants and mental diseases [121–123]. Further investigations of the roles of DISC1 in mental disorders are needed.

### TRAX

**Fig. 1:**
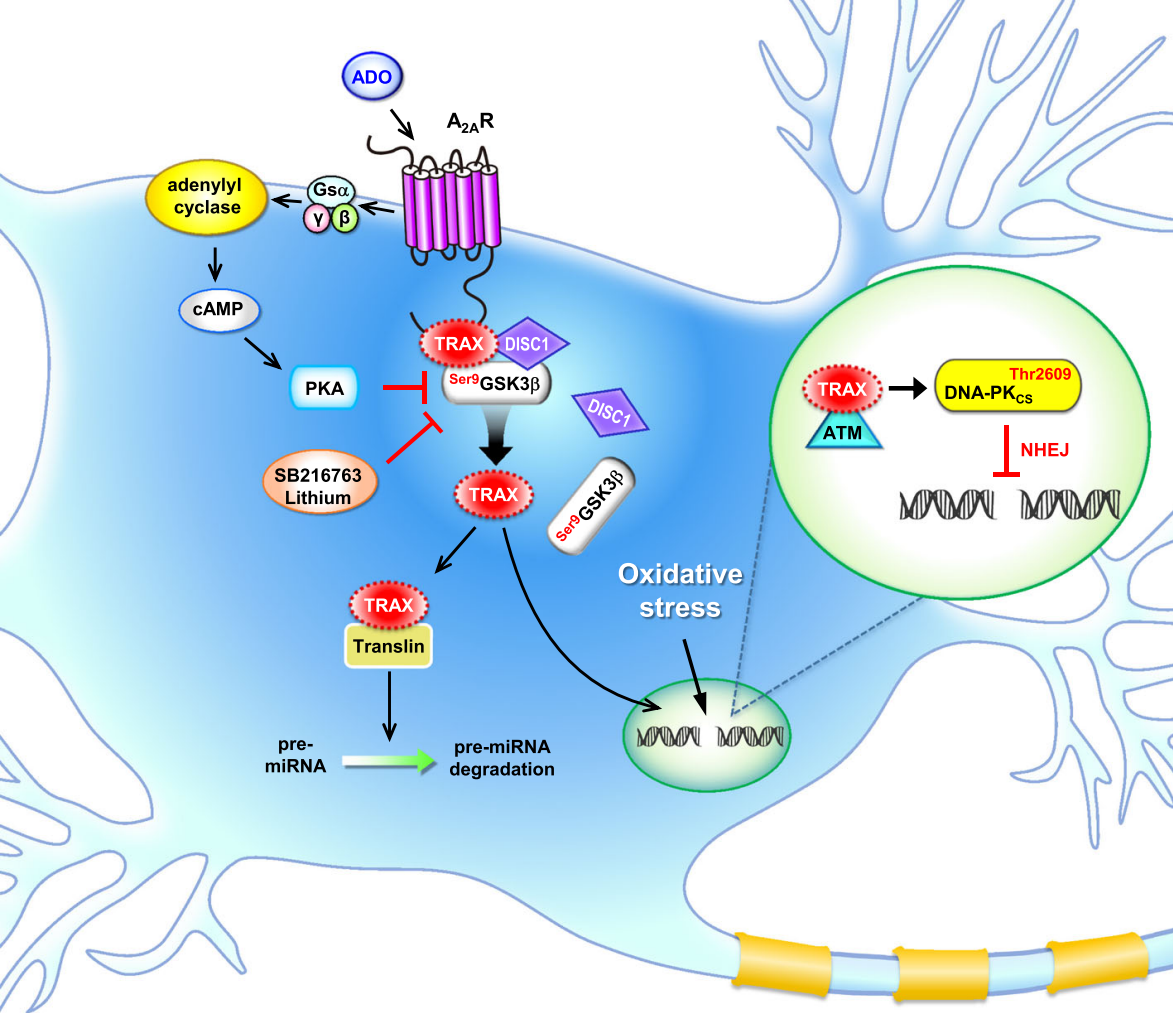
A schematic representation showing the major functions of TRAX and its interacting proteins. In neurons, TRAX interacts with the C terminus of the A_2A_ adenosine receptor (A_2A_R), a Gsα-coupled receptor that activates adenylyl cyclase to produce cAMP upon stimulation with adenosine (ADO). At the resting stage, TRAX forms complexes with GSK3β and DISC1. High oxidative stress is known to cause double-strand DNA breaks. Activating the A_2A_R/PKA-dependent pathway or inhibiting GSK3β using selective inhibitors (e.g., SB216763 or lithium) release TRAX from the complex and assist in ATM/DNA-PK-dependent non-homologous end joining (NHEJ) repair in the nuclei [5, 137]. TRAX may also bind with translin to regulate the amount of miRNA and downstream gene expression profiles [130].

TRAX was first discovered as a binding partner of translin using a yeast two-hybrid system. Amino acid sequence alignment revealed that TRAX displays 28% identity with translin [16]. Because the genetic removal of translin promotes the degradation of TRAX, TRAX stability appears to be controlled by its binding partner (i.e., translin [124]). Both TRAX and translin are highly enriched in the brain. The heteromeric complex composed of TRAX and translin shows nucleic acid binding activity in brain extracts [125] and plays a role in dendritic RNA trafficking in neurons [126, 127]. The heteromeric translin/TRAX complex also functions as an endoribonuclease that cleaves passenger strands of siRNA and therefore facilities siRNA guide strand loading onto the RNA-induced silencing complex (RISC) in *Drosophila* [128]. In contrast, the TRAX/translin complex suppresses microRNA (miRNA)-mediated silencing in mammalian cells by degrading pre-miRNA with mismatched stems and subsequently reversing miRNA-mediated silencing [129] (Fig. 1). In support of this hypothesis, TRAX/translin was recently shown to play a critical role in regulating long-term memory by suppressing microRNA silencing at activated synapses [130]. Given that aberrant profiles of miRNAs and their targeted genes have been implicated in mental disorders (such as schizophrenia, bipolar disorder and autism) (for a review, see [131]), the role of abnormal TRAX/translin regulation in mental disorders warrants future investigations.

Similar to DISC1, TRAX also has many interacting proteins with a wide variety of functions. Most of these TRAX-interacting proteins are risk genes, markers, or drug targets for psychotic disorders (e.g., schizophrenia, autism, and panic disorders; Table 1). For example, A_2A_R is the binding partner of TRAX [132]. A_2A_R is a Gsα-coupled receptor that activates the cAMP/PKA pathway upon stimulation [133]. A_2A_R activation or TRAX overexpression rescues the impaired neurite outgrowth caused by p53 blockade in a neuronal cell line (PC12) and primary hippocampal neurons. Knocking down TRAX or preventing the interaction between TRAX and its interacting protein (kinesin heavy chain member 2A, KIF2A) blocks the rescue effect of A_2A_R activation [134]. Of note, KIF2A is a schizophrenia susceptibility gene [135]. A_2A_R is a risk gene for autism and anxiety disorders, and a marker for schizophrenia (Table 1).

Two of the TRAX-interacting proteins (C1D and ataxia-telangiectasia mutated (ATM) kinase) are involved directly in DNA repair. C1D is an activator of DNA- dependent protein kinase (DNA-PK). Upon DNA damage induced by γ-irradiation, TRAX increasingly interacts with C1D in mammalian cells, suggesting that TRAX might participate in DNA repair [136]. ATM is a serine/threonine kinase and is activated and recruited by DNA double-strand breaks (DSBs) to phosphorylated proteins (e.g., histone H2A (H2AX) and p53) that are important for DNA repair. In the absence of TRAX, ATM fails to be recruited to DSB sites to initiate the DNA repair machinery and subsequently causes cell death due to insufficient DNA damage repair [5, 137]. During oxidative stress-induced DNA damage in neurons, TRAX forms a complex with DISC1 and GSK3β in the cytoplasmic region. A_2A_R stimulation activates PKA, which phosphorylates GSK3β at Ser^9^ and dissociates the TRAX/DISC1/GSK3β complex so that TRAX can enter the nuclei to facilitate DNA repair [5] (Fig. 1). The role of TRAX and its interacting proteins in mental disorders appear important because ample evidence suggests that incomplete oxidative DNA damage repair may contribute to the development of psychotic disorders [1, 17, 18]. Most of the major components (including ATM, [138, 139]) involved in TRAX-mediated DNA repair are also risk genes of mental disorders (Table 1).

Consistent with the hypothesis that TRAX is involved in the development of mental disorders, genetic studies have implicated TRAX in major psychiatric diseases. The human *TSNAX* gene is located at 1q42.1 and adjacent to the *DIS*C1 gene. Several *TSNAX* transcripts contain the *DIS*C1 sequence at the 3’ end due to intergenic splicing in human adult and fetal tissues [93]. A SNP analysis revealed that 2 SNPs (i.e., rs1615409 and rs766288) are located within intron 4 of *TSNAX*, and 2 SNPs (i.e., rs751229 and rs3738401) were found in *DISC1* in Finnish schizophrenia patients [13]. A rare AATG haplotype comprising these 4 SNPs is positively associated with the reaction time to visual targets and negatively with the gray matter density in Finnish schizophrenia patients [140]. Furthermore, another SNP analysis identified that rs1655285 at intron 5 of *TSNAX* and a haplotype comprising rs1630250 and rs1615409 within *TSNAX* are associated with Finnish bipolar spectrum disorder [15]. The SNP rs766288 at intron 4 of *TSNAX* has been reported to be associated with Japanese female major depressive disorder [14]. These studies collectively suggest that TRAX is a risk gene for major mental diseases. It should also be noted that TRAX and DISC1 share several interacting proteins (e.g., GSK3β and AKAP9; Table 2) and functional pathways/machineries (e.g., the cAMP/PKA pathway, Wnt signaling, intracellular transport and DNA repair; Table 2); thus, they may act together to regulate important pathophysiological events, including the development of mental disorders.

### Regulation of the TRAX/DISC1/GSK3β complex and therapeutic relevance

Although DISC1 mediates many different cellular functions, it has not been implicated in DNA repair until a recent report [5] demonstrating that DISC1 interacts with GSK3β and TRAX; this complex facilitates DNA repair by binding to ATM [137]. This finding leads to a new mechanistic role of DISC1 in mental disorders in which accumulating oxidative DNA damage and insufficient DNA repair contribute to the pathogenesis [1, 17, 18]. Disassembly of the TRAX/DISC1/GSK3β complex, followed by the release of TRAX, provides a new means to facilitate DNA repair and ameliorate the damage caused by unrepaired DSBs. For example, A_2A_R activation dissociates TRAX/DISC1/GSK3β complex tethering at its C terminus through a PKA-dependent pathway and amends the DNA damage-induced apoptosis [5]. Consistent with an important role of A_2A_R in facilitating DNA repair, A_2A_R activation ameliorates oxidative DNA damage in human medium spiny neurons (MSNs) derived from induced pluripotent stem cells (iPSCs) [141]. Interestingly, the amount of A_2A_R is altered in different brain regions of patients with schizophrenia [142, 143], supporting that A_2A_R might play an important role in schizophrenia. Because A_2A_R is an antagonistic binding partner of the D2R and may suppress the hyperfunction of D2R in schizophrenia [144], A_2A_R agonists are potentially advantageous anti-schizophrenic drugs (for a review, see [145]). D2R is a primary target of antipsychotic drugs. It forms complex with not only A_2A_R but also DISC1 to mediate the D2R-dependent activation of GSK3β [146]. This is of great interest because DISC1 binds with TRAX and GSK3β [5]. Whether D2R activation affects the accumulation of oxidative DNA damage and contributes to pathogenesis requires further investigation.

It is important to note that adenosine is known to regulate the dopamine and glutamate- mediated neurotransmissions, the major neurotransmitter systems involved in schizophrenia pathophysiology [147–149]. Dysfunction of purinergic system is one of the factors that cause schizophrenia [149, 150]. Moreover, inhibition of adenosine kinase (ADK), which controls adenosine level, exhibits anti-psychotic-like efficacy, while overexpression of ADK causes changes in the sensitivity to psychomimetic drugs in mice [151, 152]. Consistent with the abovementioned hypothesis, increased brain adenosine tone using an inhibitor of adenosine uptake (i.e., dipyridamole) indirectly activates adenosine receptors and hasWr beneficial effects on patients with schizophrenia [153]. Likewise, inhibiting adenosine clearance using ABT702 to globally increase adenosine tone also ameliorates the psychotic and cognitive phenotypes of schizophrenia in mice [151]. Given that adenosine is also known to play an important role during neurodevelopment. (for a review, see [154]). Augmenting the adenosine tone in the brain using various approaches might thus serve as a therapeutic means to treat schizophrenia as well as to prevent the development of schizophrenia [154, 155].

Lithium is an inhibitor of GSK3 and a common mood stabilizer for treating mental disorders. To date, the underlying molecular mechanism of lithium’s action remains largely elusive [156, 157]. Accumulating evidence suggests that chronic treatment with lithium inhibits the oxidative damage evoked by glutamate [158] and increases the expression level of the anti-apoptotic factor Bcl2 [159, 160]. Treatment with lithium also protects neurons by facilitating the NHEJ repair-mediated DNA repair pathway [161]. Chronic treatment with lithium results in not only the inhibition of GSK3β but also the regulation of many anti-apoptotic proteins. For example, lithium inhibits calcium influx via regulating the NMDA receptor and reduces apoptosis by directly inhibiting GSK3β [162]. Because inhibiting GSK3β causes the disassembly of the TRAX/DISC1/GSK3β complex and releases TRAX to facilitate DNA repair [5], at least part of the actions of lithium might be mediated by the TRAX/DISC1/GSK3β complex. New GSK3β inhibitors have been actively developed for brain diseases [163], which may pave the way for establishing new treatments for schizophrenia.

## Conclusions

As a major gene implicated in schizophrenia and other mental disorders, DISC1 is known to regulate various cellular functions by interacting with proteins of different machineries. Ample evidence suggests that DISC1 is a hub protein for schizophrenia and possibly other mental diseases. Emerging evidence also suggests that TRAX, a neighboring gene of DISC1, not only physically interacts with DISC1 but also switches binding partners under different pathophysiological conditions as does DISC1. Because the studies regarding TRAX are still in their infancy, the overlapping functional pathways of DISC1 and TRAX appear limited at this time (Table 2) but may become more evident when more binding partners of TRAX are revealed in the future. Most importantly, genetic evidence suggests that DISC1 and TRAX are closely associated with several major mental disorders (such as schizophrenia, autism, and anxiety disorder; Table 1). Therefore, it is certainly worth further exploring the role of the DISC1/TRAX complex in psychiatric disorders. Because oxidative DNA damage accumulation and insufficient DNA repair have been implicated in the development and progression of mental disorders, the recently reported function of the DISC1/TRAX/GSK3β complex in DNA repair also warrants further investigations of the temple and the special regulation of this complex during neuronal development and disease progression. Further understanding of when and where the DISC1/TRAX/GSK3β complex is formed and how the complex can be effectively dissembled by either GSK3β inhibitors or PKA activators (such as A_2A_R agonists or PDE4 inhibitors) would pave the way for developing new therapeutic agents for mental disorders.

## Abbreviations

8-OHdG: 8-hydroxy-2'-deoxyguanosine
A_2a_R: A_2A_ adenosine receptor
ADK: Adenosine kinase
ADO: Adenosine
AKAP9: A-kinase anchoring protein 9
AMPA: α-amino-3-hydroxy-5-methyl-4- isoxazolepropionic acid
ATF: Activating transcription factor
ATM: Ataxia-telangiectasia mutated
BBS4: Bardet-Biedl syndrome 4
C1D: Nuclear matrix protein C1D
cAMP: Cyclic adenosine monophosphate
CREB: Calcium/calmodulin-dependent protein kinase II (CaMKII)/CaMKIV-cAMP response element binding protein
CRMP-2: Collapsin response mediator protein-2
D2R: D2 Dopamine receptor
DG: Dentate gyrus
DISC1: Disrupted in schizophrenia 1
DIXDC1: DIX domain containing 1
DNA-PK: DNA- dependent protein kinase
DSBs: DNA double-strand breaks
ETC: Electron transport chain
FEZ1: Fasciculation and elongation protein zeta 1
GSK3: Glycogen synthase kinase 3
H2AX: Histone H2A
HO-1: Heme oxygenase-1
hOGG1: Human 8-oxoguanine DNA N-glycosylase 1
HZF: Hematopoietic zinc finger
iPSCs: Induced pluripotent stem cells
ITRP1: Inositol-1,4,5-trisphosphate receptor type 1
kal-7: Kalirin-7
KIF2A: Kinesin heavy chain member 2A
LIS1: Lissencephaly 1
LTD: Long-term depression
LTP: Long-term potentiation
MAPs: Microtubule-associated proteins
MEA2: Male-enhanced antigen 2
miRNA: microRNA
Miro1/2: Mitochondrial Rho GTPase 1/2
MSNs: Medium spiny neurons
MYD88: Myeloid differentiation primary response gene 88
NDEL1: Nuclear distribution nudE-Like 1
NDUFV2: NADH:Ubiquinone oxidoreductase core subunit V2
NHEJ: Non-homologous end joining
NMDA: N-methyl-D-aspartate
NRF2: Nuclear-localized NF-E2-related factor 2
PBMCs: Peripheral blood mononuclear cells
PCM1: Pericentriolar material 1
PCP: Phencyclidine
PDE4B: Phosphodiesterase 4B
PKA: Protein kinase A
PLCβ1: Phospholipase C Beta 1
PSD: Post-synaptic density
Rac1: Rac family small GTPase 1
Rad21: Double-strand-break repair protein rad21 homolog
RISC: RNA-induced silencing complex
ROS: Reactive oxygen species
SNPH: Syntaphilin
SUN1: SUN domain-containing protein 1
SYT-1: Synaptotagmin-1
TLR3: Toll-like receptor 3
TNIK: TRAF2 and NCK interacting kinase
TRAK1/2: Trafficking kinesin protein-1/2
TRAX: Translin-associated factor X
WNT3A: Wnt family member 3A
XPD: Xeroderma pigmentosum group D
XRCC: X-ray repair cross complementing
